# Quantitative Analysis of Single Nucleotide Polymorphisms within Copy Number Variation

**DOI:** 10.1371/journal.pone.0003906

**Published:** 2008-12-18

**Authors:** Soohyun Lee, Simon Kasif, Zhiping Weng, Charles R. Cantor

**Affiliations:** 1 Bioinformatics Program, Boston University, Boston, Massachusetts, United States of America; 2 Department of Biomedical Engineering, Boston University, Boston, Massachusetts, United States of America; 3 Children's Hospital Informatics Program at Harvard-MIT Health Sciences and Technology, Boston, Massachusetts, United States of America; 4 Sequenom Inc., San Diego, California, United States of America; 5 Biochemistry and Molecular Pharmacology, University of Massachusetts, Worcester, Massachusetts, United States of America; Columbia University, United States of America

## Abstract

**Background:**

Single nucleotide polymorphisms (SNPs) have been used extensively in genetics and epidemiology studies. Traditionally, SNPs that did not pass the Hardy-Weinberg equilibrium (HWE) test were excluded from these analyses. Many investigators have addressed possible causes for departure from HWE, including genotyping errors, population admixture and segmental duplication. Recent large-scale surveys have revealed abundant structural variations in the human genome, including copy number variations (CNVs). This suggests that a significant number of SNPs must be within these regions, which may cause deviation from HWE.

**Results:**

We performed a Bayesian analysis on the potential effect of copy number variation, segmental duplication and genotyping errors on the behavior of SNPs. Our results suggest that copy number variation is a major factor of HWE violation for SNPs with a small minor allele frequency, when the sample size is large and the genotyping error rate is 0∼1%.

**Conclusions:**

Our study provides the posterior probability that a SNP falls in a CNV or a segmental duplication, given the observed allele frequency of the SNP, sample size and the significance level of HWE testing.

## Introduction

### 

#### 1. Single nucleotide polymorphism (SNP) and Hardy-Weinberg equilibrium (HWE)

Single nucleotide polymorphisms (SNPs) are common biallelic variations that are widely used as genetic markers in linkage analyses and association studies[1]. Most human SNPs satisfy the Hardy-Weinberg equilibrium (HWE), the condition of allelic independence, in which allele frequencies and genotype frequencies do not change over generations[2], [3]. Hunter et al.[4] reported that 5.0% and 1.3% of SNPs in their analysis deviated from HWE, at significance level α = 0.05 and α = 0.01, respectively, which indicates that most of the human SNPs are under the null hypothesis of HWE. A departure from HWE can be explained by natural selection, population admixture, inbreeding, experimental errors and duplication[5]. Conventionally SNPs that are significantly deviated from HWE are discarded before further analysis.

#### 2. Copy number variation (CNV) and segmental duplication (SD)

A copy number variation (CNV) is a genomic segment larger than 1 kb that occurs in variable numbers in the genome. When the variant frequency is larger than 1% in a population, it is called a copy number polymorphism (CNP). In some contexts, CNV stands for copy number variants[6], which refers to individuals whose copy number is different from the majority in a population. Here, by CNV we refer to a specific locus, or a genetic marker in a population that shows variations among individuals.

A segmental duplication (SD) refers to a large duplicated sequence in the genome, conventionally longer than 1 kb with at least 90% sequence identity between duplicate copies (reviewed by Bailey and Eichler[7]). SDs occupy about 5% of the human genome[8]. SDs are closely related to CNVs, except that an SD does not have a varying copy number within a population. Based on a single Caucasian individual's diploid genome sequence that came out recently, about 55% of CNVs seem to overlap with an annotated SD[9]. A similar rate of overlap had been reported in another study based on comparison between the human genome reference sequence and a fosmid-paired-end library[10]. Redon et al.[11] suggested that the significant overlap between SD and CNV is partly because of incorrect annotation of CNVs as SDs; i.e. the number of individuals sequences was not large enough to detect rare variants. Moreover, CNVs and SDs can be viewed as a special case of one another. Sebat et al.[12] viewed copy number gains as recent segmental duplications. We adopt a view that SD is an extreme case of CNV in which duplication frequency is 100%.

#### 3. SNPs in a CNV

Recent studies show that at least 12%–15% of the human genome is covered by copy number variations[11], [12]. Moreover, 56% of the CNVs identified were in known genes, according to Iafrate et al.[13] and Zogopoulos et al.[14]. The large proportion of CNVs in the genome indicates that a significant number of SNPs may fall in these regions. Nguyen et al. showed that SNPs are significantly enriched in known human CNVs[15].

We are interested to know how a SNP would behave when it is in a copy number variation. We begin with an ‘observed SNP’ site, that shows two different bases in sequencing or genotyping experiments. The measured genotype and allele frequencies of an observed SNP may not reflect the true frequencies when additional copies exist. An observed SNP may not even be a true SNP, but instead a variation between two duplicate copies.

It is difficult to separate duplicate copies experimentally. The sequences flanking the two loci are nearly identical and PCR (polymerase chain reaction) and extension reactions cannot differentiate them. Finding out the exact genotypes for CNVs is also a challenging problem and only relative quantification is available to date[16]. Thus, computational inference can be useful at this point, for understanding the HWD of SNPs in a CNV.

Our study focused on relatively small scale SNP studies with limited information. Detection and validation of CNVs through experimental and computational methods have been an ongoing problem. However such information is often limited due to difference in population (e.g. ethnicity), lack of confirmed boundaries, and quantification relative to the population average than the absolute number of copies.

Methods have been developed specifically for detecting CNVs using a large number of SNPs. SNP arrays (BeadArray™ by Illumina and GeneChip® by Affymetrix) became available recently that allow simultaneous genotyping of CNVs and SNPs. Software that detects CNVs from the SNP arrays has been developed (eg. BeadStudio LOH+ by Illumina and QuantiSNP by Colella et al.[17]). QuantiSNP uses the information that many consecutive SNPs within a CNV region must share the effect of a CNV and has an estimated false positive rate of 1 CNV in 100,000 SNPs. McCaroll et al.[18] identified 541 deletion variants by using the neighboring-marker effect as well as HWD and non-Mendelian inheritance. Most of these approaches use the logic that closely located neighboring SNPs share the same CNV.

However, not every investigator genotypes such a dense set of SNPs, depending on the goal of the genetics or epidemiology study. Closely positioned SNPs are often in linkage disequilibrium and many investigators prefer typing distant SNPs for cost effectiveness. Our goal is to compute the theoretical degree of contribution of CNVs and SDs to HWD of individual SNPs provided limited knowledge of CNVs in the particular population under study, rather than developing a method of detecting CNVs using a dense set of genotyped SNPs.

The power to detect deviation from HWE in SNPs in a segmentally duplicated region was recently examined by theoretical analysis and simulation[19]. Here we provide a more general model that considers CNVs and their relative contribution to HWD. We construct a quantitative SNP-CNV mixture model and present Bayesian estimates of probability of a SNP being in a CNV, given that it is significantly deviated from HWE. To our knowledge this is the first study to provide the posterior probabilities P(CNV|HWD).

## Results

### I. Model and assumptions

According to Redon et al., only about 1∼2% of CNVs are multi-allelic and 5∼10% are complex[11]. Thus, the majority of the CNVs detected may be biallelic, which involves either a single duplication or a single deletion. It is relatively easier to identify deletion polymorphisms, by null allele individuals. Assuming that there is no null-allele individual, we propose that a biallelic CNV assumption is a good start for quantitative modeling. An extension may be applied to multiallelic or more complex cases. In order to deal with multiallelic CNVs, more parametric assumptions are required such as how sequence variations are distributed across different copies. We believe that a multiallelic extension may be more informative after we gain more knowledge about these parameters.

**Figure 1:**
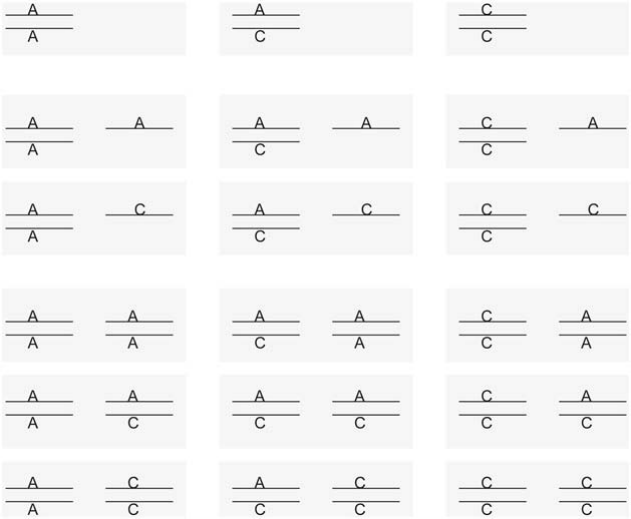
Possible cases of a SNP in a biallelic CNV. All possible cases of observed SNPs on a biallelic, duplication-type CNV. Each gray box represents an individual. Two parallel lines are homologous chromosomes. The left homologous pair represents the original site (L1) and the right pair represents the ectopic site (L2). The ectopic site may not exist or exist in only one of the homologous chromosomes in some individuals.

Under a biallelic CNV assumption, we can imagine a situation as depicted in Figure 1. Suppose that we have two sites L1 and L2, where L1 is always a diploid and L2 is a variable ectopic site. In some individuals, L2 may not exist or exist in only one of the two homologous chromosomes. Suppose the observed SNP has alleles A and C, with A as the minor allele, as an example. Each of the two sites can be either heterozygotic or monomorphic. We denote by p_1_ the true frequency of allele A at L1, and by p_2_ the true frequency of allele A at L2. Though we assume that A is the observed minor allele, it does not have to be a minor allele at each site and p_1_ and p_2_ may range from 0 to 1. Additionally, we introduce a new parameter r, the frequency of having both sites L1 and L2, as apposed to having only L1. Thus, r refers to the true allele frequency of the underlying CNV. For a CNV, r can vary between 0 and 1. When there is no duplication (i.e. regular genomic regions), r = 0. When duplication is fixed in all individuals in the population (segmental duplication), r = 1. For convenience, here r∈(0,1) (i.e. 0<r<1) is treated equivalent to a CNV, r = 0 to a regular genomic region, and r = 1 to a SD.

If both sites are polymorphic with different pairs of bases, the observed SNP will be triallelic (or even quadrallelic), which are not considered in the current study. Here, we assume the observed SNP is biallelic, as well as the true sites and the CNV itself.

#### Theoretical derivation of observed genotype frequencies

Given true SNP allele frequencies p_1_ and p_2_ and CNV allele frequency r, observed SNP genotype frequencies 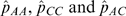 were derived, under the assumption that each of the three markers (two SNP sites and a CNV) is independent and under Hardy-Weinberg equilibrium (details in Method S1):
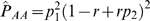
(1)

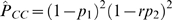
(2)

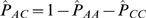
(3)Observed allele frequencies can be directly calculated from observed genotype frequencies.
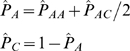
(4)


#### SNP genotyping errors

In theory, SNP genotyping errors can be in both ways and its rate depends on which nucleotides are involved. However, it is more common to misread a heterozygote as a homozygote. In our mixture model, we take a conservative approach and assume that all genotyping errors mistake a heterozygote as a homozygote, and not the other way around. If we consider both directions, the two effects counterbalance each other and contribute less to HWD. Thus, our assumption of one-way genotyping error means that the genotyping error fully contributes to HWD and does not cancel out within itself.

### II. Effect of allele frequency parameters on HWD

#### 1. Measure of HWD

Our first goal is to understand the relationship between HWD, r, p_1,_ p_2_ and 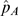. For this purpose, we used a quantitative measure of HWD. A measure of Hardy-Weinberg disequilibrium, θ, has been suggested by Olson and Foley[20].


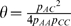
, where 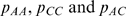 are frequencies of genotypes AA, CC and AC.

Under HWE, θ = 1. When there are excessive heterozygotes, θ>1. When there are more homozygotes than expected under HWE, θ<1. Unlike other HWD measures such as the disequilibrium parameter D[21] and the inbreeding coefficient *f* [22], θ does not assume symmetric deviations from the two homozygote frequencies, which is useful for our analysis because the effect of a CNV on the two homozygote frequencies is not always symmetric.

#### 2. Behavior of θ with respect to r, p_1_ and p_2_

**Figure 2:**
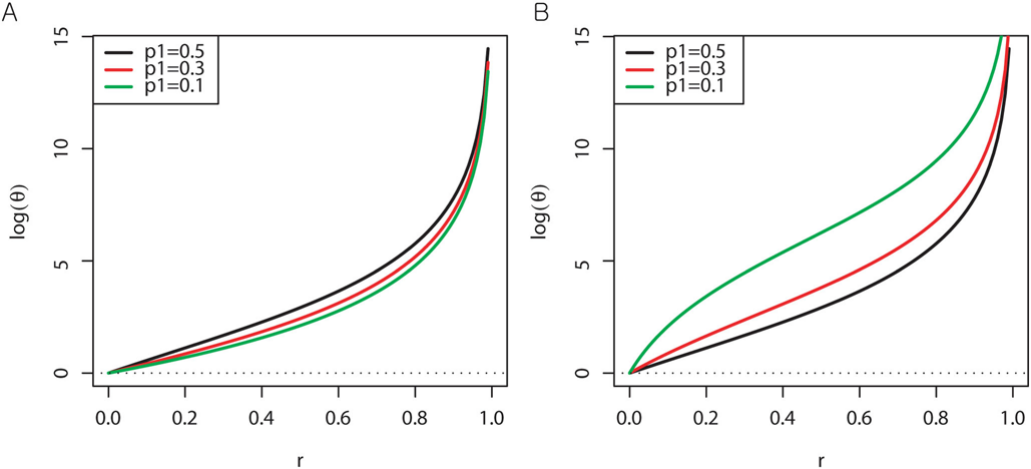
r vs log(θ), given true allele frequency. A. p_2_ = 0, B. p_2_ = 1. Log base 2.

As seen in Figure 2, θ monotonically increases with r, regardless of p_1_ and p_2_. This indicates that the ectopic site contributes to increasing the number of observed heterozygotes relative to homozygotes. Based on the assumption of no other causes of HWD such as SNP genotyping errors, θ never goes below 1 (log(θ) is always ≥0). Thus, duplication always results in excessive heterozygotes.

#### 3. Estimation of r, given θ and an observed minor allele frequency

**Figure 3:**
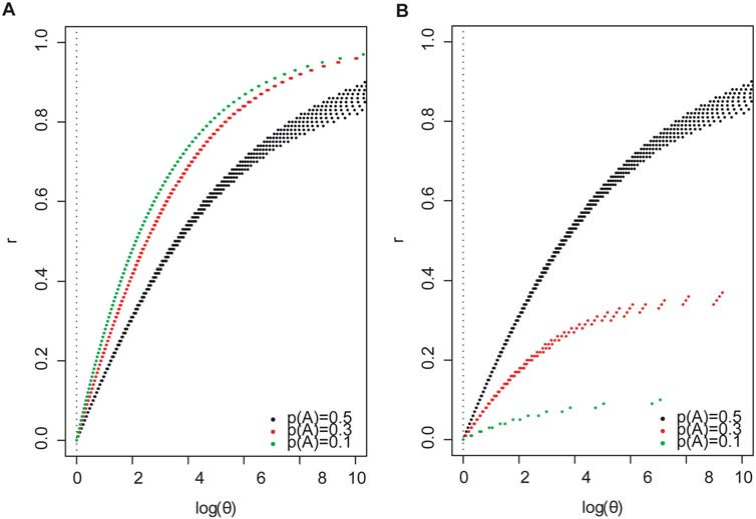
log(θ) vs r, given observed allele frequency. A. p_2_ = 0, B. p_2_ = 1. Log base 2. Observed allele frequencies are derived from computed observed genotype frequencies.

Given the observed minor allele frequency, the possible values of r vary widely depending on the assumption of p_2_. The plots in Figure 3 were drawn based on the simulation described above. A larger θ always indicates a larger r, given 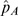. A higher 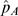 may indicate a larger or a smaller r, depending on p_2_.

#### 4. Range of p_1_, given an observed allele frequency and r

**Figure 4:**
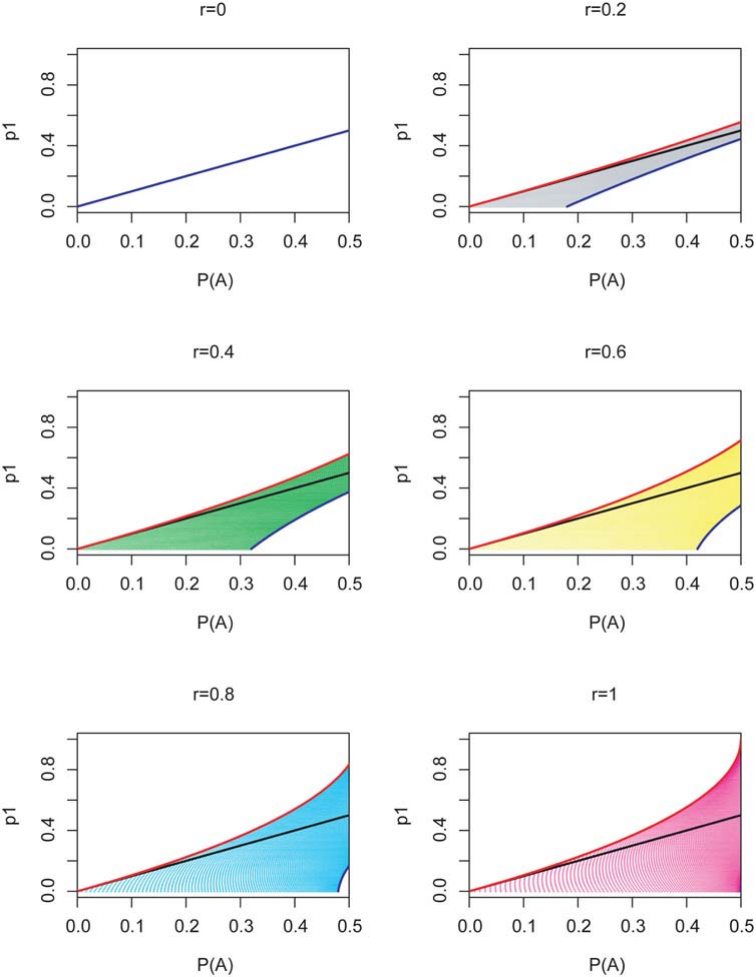
Range of p_1_, given r and 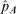. The black diagonal line is the case where the true frequency p_1_ is identical to the observed frequency. Red and blue curves represent p_2_ = 0 and p_2_ = 1, respectively.

Figure 4 shows the relationship between the true and the observed allele frequencies given r. When r is large and the minor allele frequency is large, the deviation of observed allele frequency from true allele frequency p_1_ can be very large. Thus, in this case the observed allele frequency cannot serve as a substitute for the true allele frequency. In the majority of the cases, the minor allele frequency is overestimated. Figure S1 shows the range of true allele frequency given pooled sample allele frequencies.

### III. Probability that an HWE-violating SNP is in a CNV

P(CNV|HWD), or the probability that a SNP is in a CNV (i.e. r∈(0,1)), given that the SNP is in HWD, was computed at different observed allele frequency(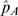), significance level for HWD testing (α), sample size (n) and SNP genotyping error (e_g_). Several hypothesis tests for HWE have been proposed, including the most commonly used chi-square goodness-of-fit test[23]. Here we used a chi-square test. We used two different prior distributions for true CNV allele frequency r; uniform and beta distributions. The uniform prior assumes equal probability density for all allele frequency, whereas the beta distribution assumes higher probability towards a smaller r (more detail can be found in the discussion section and Method S1).

**Figure 5:**
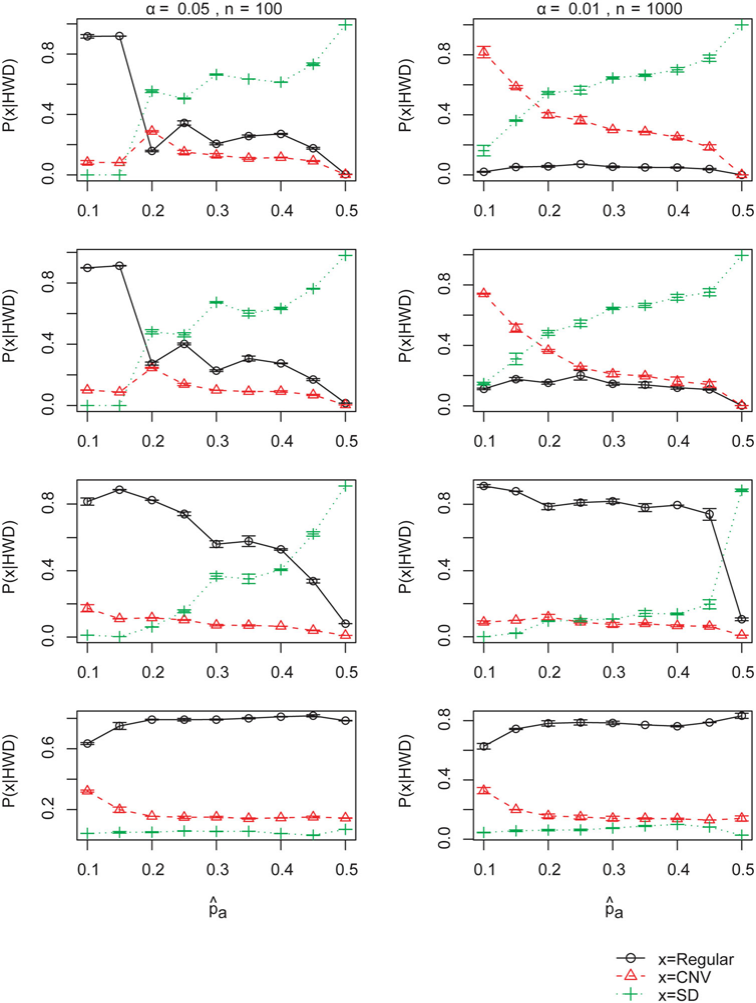
Posterior probabilities given HWD. The posterior probabilities given HWD computed using the beta prior, at n = 100, α = 0.05 (left), and n = 1000, α = 0.01 (right), with respect to observed allele frequency 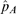. Each row respresents error rate of 0%, 1%, 5% and 25%, from top to bottom, respectively. Estimates are the sample mean of two replicates and the standard deviations are depicted with error bars.

As seen in Figure 5, at α = 0.05 and n = 100, under the assumption of no genotyping error and a beta prior, segmental duplication (r = 1) was the most responsible cause of HWD. Interestingly, when the observed minor allele frequency is small (<0.2), duplicons happen to generate allele frequencies that mimic apparent HWE, and random variation is the most important cause of HWD at these small minor allele frequencies. Under the beta prior with 5% genotyping error, the contribution from SD or CNV becomes minor, except at 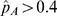. Under a very large genotyping error, the probability of the SNP not being in a CNV or SD is 60∼80%. In general, a 1% Genotyping error made little difference compared to the case of no genotyping error. For n = 1000 and α = 0.01, with 0∼1% genotyping errors, the most likely cause of HWD was CNV or SD, depending on the observed allele frequency. CNV and SD tend to counterbalance one-way genotyping errors, as seen clearly in the case of a 25% error rate.

**Figure 6:**
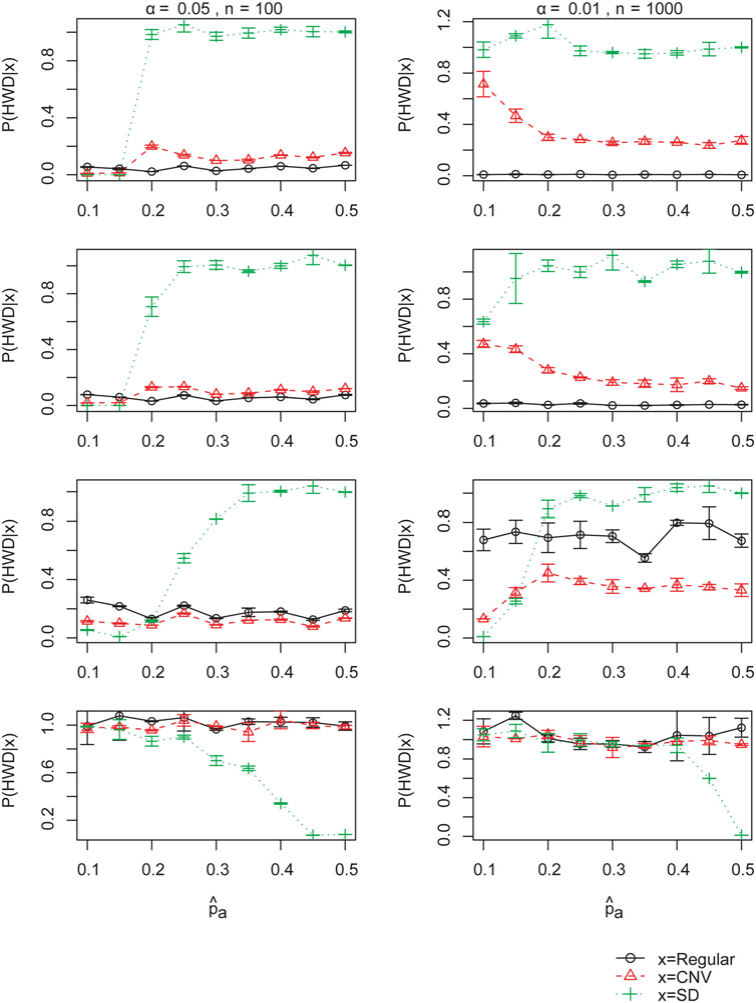
Likelihoods of HWD. The likelihoods of HWD computed using the beta prior, at n = 100, α = 0.05 (left), and n = 1000, α = 0.01 (right), with respect to observed allele frequency 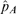. Each row respresents error rate of 0%, 1%, 5% and 25%, from top to bottom, respectively. Estimates are the sample mean of two replicates and the standard deviations are depicted with error bars.

The relative contribution by duplication is quite different depending on the stringency of HWD testing (Figure 5, No genotyping error). At α = 0.05, theoretically about 5% of SNPs in the regular regions must be determined to be in HWD, whereas at α = 0.01, only 1% contributes to HWD. Also at α = 0.05 and n = 100, SNPs in duplicons (CNV/SD) often do not generate a sufficient deviation from HWE to be detected by the testing, whereas at α = 0.01 and n = 1000, the likelihood of HWD given CNV or SD become much larger (Figure 6, No genotyping error) that the posterior probabilities point to CNVs and SDs as a major contributor to HWD.

The uniform model (Figure S2, S3) tends to conclude a higher contribution of CNV to HWD compared to the beta model, which is intuitive because the uniform model assumes more CNVs whose allele frequencies are close to SD than to regular regions.

The computation by sampling directly from priors converged, as suggested by one of the cases shown here (Figure 6). The computation was done by summing the probabilities of different cases of r, p_1_ and p_2_. Some individual cases failed to converge but did not affect the overall summation, because the values were ignorably small (Figure S4).

## Discussion

### Effect of allele frequency parameters on HWD

Our simulation shows that the HWD measure θ only increases with respect to r under no experimental errors, supporting that duplication acts in the direction of increasing observed heterozygotes.

### Probability that an HWE-violating SNP is in a CNV

Our results suggest that copy number variation can be a major contributor to HWD, even assuming the tendency towards small variant frequencies of CNV, especially at a low observed SNP minor allele frequency and large sample size. Segmental duplication is a major effect at a higher observed SNP minor allele frequency. About 1% genotyping errors did not make much difference to P(CNV|HWD). At a 5% or higher genotyping error, CNV or SD is less likely to be the cause of HWD.

Out results show that the probability of a SNP being in a duplicated region given HWD depends on the observed allele frequency. In case of a high observed minor allele frequency, HWD tends to be due to duplication, whereas in case of a small 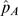, HWD is mainly due to SNP genotyping error and random variation. This is mainly because the effect of duplication can be buffered for low observed minor allele frequencies.

Hosking et al.[24] analyzed 36 HWE-violating SNPs and concluded that 58% of these cases were due to genotyping errors. This is an average that does not depend on observed minor allele frequency, but it is consistent with our result with a 5% genotyping-error model. The authors found 14% ‘non-specific’ cases where a primer/probe set can bind to multiple regions in the genome. These 14% may be included either in annotated segmental duplication or copy number variation. The other 28% showed no reason for HWD. Some of these cases may belong to a previously unannotated SD or CNV.

### Prior knowledge of r

For the prior distribution of r, we incorporated estimates from previous studies about CNVs. Fredman et al.[25] estimated through an *in silico* analysis that 3.7% of validated SNPs and 13.1% of nonvalidated SNPs were found in segmental duplicons. We interpret this as 7% on average, considering 65.2% of the SNPs used were valid in their analysis. It is similar to but slightly higher than the estimated proportion of SD in the genome. We simply used the reported genomic proportions of CNV or of SD as the prior probabilities of a SNP being in a CNV or an SD. Considering the previous reports[15] that SNPs are enriched in CNVs, using the genomic proportions as a prior probability is conservative in estimating the posterior probability of CNVs and SDs.

Our beta prior assumes about 50% of the CNVs have a minor allele frequency (MAF) more than 3.5% and about 13% and 1.5% have >10% and >20% MAFs, respectively, which are approximately consistent with Iafrate et al.'s estimate[13]. 12% of the CNVs identified by Iafrate et al. had >10% MAF and 3% had frequency of >20%. More conservative estimates have been reported as well. A recent study using about 1200 North American individuals estimated that more than 93% of CNV regions (CNVRs) have less than 1% MAF. Only 1% of the CNVRs analyzed had MAF >5%. The authors suggested that CNVs are not likely to affect SNP association studies seriously because of the low MAF. According to these recent estimates, a more realistic prior distribution of r would be even more skewed than the beta distribution that we have used. Another recent study by Wong et al.[16] detected 3,654 CNVs and 800 of them had at least 3% frequency, indicating a higher estimate for CNV minor allele frequencies.

### SNP genotyping errors

Genotyping error rates for Sequenom (San Diego, California, USA), Illumina (San Diego, California, USA) and other new methods were reported as less than 1% (personal communication, Cantor). Sources and types of genotyping errors may vary and such heterogeneous effects were not considered in our model.

Cox and Kraft[5] showed that HWE tests have low power in detecting genotyping errors. This means that most of the genotyping errors do not cause departure from HWE. Our study indicates that once a SNP violates HWE, there is a good chance to have genotyping errors as well as segmental duplication or copy number variation, when the genotyping error is above 5%. These two results are not contradictory but provide different angles. As seen in the likelihood of HWD given no CNV or SD (Figure 6), the sensitivity of detecting genotyping errors using HWD is very low. However, the relative contribution of genotyping error can become large when other factors are even less likely to cause HWD.

### HWE violation and association studies

Hunter et al.[4] proposed to include HWE-deviated SNPs in case-control association studies because association tests do not assume HWE. Trikalinos et al.[26], however, showed through a meta-analysis of 591 previous association studies that HWE-violating samples gave in significantly different results in the association testing. Taken together, we'd like to adopt a view that the association tests do not assume HWE, but can be affected by HWD, partly because these tests do assume that the SNPs are not in duplicated regions. Thus, it seems useful to know the effect of duplication on the HWE of a SNP.

### Independence and HWE assumptions

Although at least some CNVs are generated in tandem[13], [15], the extent to which tandem and interspersed duplications contribute to the entire CNV space is unknown. As for segmental duplication, 45% and 47% are tandem and interchromosomal, respectively, in humans[7], indicating the possibility of abundant interspersed CNVs. Our assumption of independence between duplicate sites may not hold if they are tandem and in linkage disequilibrium.

In addition, we assumed that an underlying CNV itself is under HWE. Sebat et al.[12] suggests that CNVs might be under negative selection. A recent survey on experimentally identified CNVs by Nguyen et al.[15] revealed that human CNVs are significantly enriched in telomeric and centromeric regions and protein coding genes, indicating nonneutral evolution of CNVs. However, the extent to which such selective pressures can affect the HWE of a CNV has yet to be studied.

Nguyen et al.[15] also revealed that CNVs are associated with high synonymous and nonsynonymous substitution rates, indicating that the assumption of a constant SNP rate on duplicated and nonduplicated regions may not hold. Other factors may also affect the priors for SNP allele frequencies, including nonuniform allele frequency distribution and gene conversion[27], [28].

Our model assumes duplication, genotyping error and random variation as the only sources of HWD. In reality, there are other sources of HWD. One of them is the noise in the actual population. Shoemaker et al.[29] noted that a population is not under a perfect Hardy-Weinberg equilibrium. In their analysis, the authors used inbreeding coefficient f_A_<|0.03| as the limit of HWD in human population, as suggested by a National Research Council report (National Research Council 1996)[30]. The inbreeding coefficient is one of the proposed measures of HWD and f_A_ = 0 indicates HWE[22]. Our study assumes that the population is under the perfect HWE in each locus. Sampling of individuals in real experiments is not perfectly random and can be another source of bias.

### Population admixture

Our model does not consider population admixture effect. Population admixture is an important confounding factor in case-control studies and it is known that the admixture effect causes deviation from HWE, as we mentioned in the background section of our manuscript. Nevertheless, with sample size <1000, population admixture can be detected by HWE testing only when f>0.4 and k>0.2, where f is the allele frequency difference between the mixed populations and k the proportion of the minor population[31]. A recent study indicates that most populations do not satisfy this criterion[32]. Thus, we assume that population admixture has minor effect on HWE in most populations. It would be helpful to incorporate admixture effect to our model, once we obtain sufficient knowledge about the degree of population-difference of CNVs. Our study focuses on the relative contribution of genotyping errors and duplication effect.

### Conclusions

Our study shows that the degree of HWD increases with respect to r, the frequency of two-copy alleles. Duplication acts in the direction of increasing observed heterozygotes. The results of our Bayesian analysis suggest that copy number variation can be a major contributor to HWD, when sample size is large and genotyping error is small. The relative contribution of CNV and SD to HWD varies with observed SNP allele frequency.

## Materials and Methods

### I. Effect of allele frequency parameters on HWD

#### 1. Relationship between r and θ

We varied r, p_1_ and p_2_ and observed genotype frequencies and allele frequencies were computed. Values for log_2_(θ) were also obtained from the computed genotype frequencies. The simulation was done using a Perl script that we wrote, and the plots were drawn using the R language.

#### 2. Range of p_1_, given observed allele frequency and r

Given a value of r, either estimated or derived from genotyping experiments, we asked whether the true allele frequency for the SNP could be derived. For varying values of p_2_, we have plotted the range of possible values of the true allele frequency p_1_, given observed allele frequency 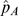.

Here, the range of p_1_ is not less informative than a posterior distribution of p_1_, because in this case the posterior probability depends only on p_2_, for which we assumed a uniform prior except in marginal cases.


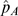
 can be expressed in a closed form in terms of r, p_1_ and p_2_. When r is fixed, p_1_ and p_2_ have complementary effect on 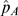. Thus, the maximum and minimum possible values of p_1_ can be obtained by assuming the minimum and maximum values of p_2_, given r and 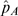. Figure 4 illustrates the range of p_1_ for different values of r and 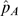. The plots were generated by computing 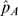 from discretized p_1_, p_2_ values ranging from 0 to 1, for a given r.

Additionally, we have looked at the range of p_1_, given the observed allele frequency measured using a pooled-sample technique. The pooled-sample SNP allele frequency, which is different from the allele frequency derived from genotype frequencies (equations (4)), can also be expressed in terms of r, p_1_ and p_2_:
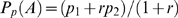
(5)


Experimentally, a pooled sample allele frequency can be obtained by pooling DNA samples and measuring the relative quantities of each allele in the pooled sample[33]. Ideally this measure is identical to the allele frequency calculated from the genotype frequencies. However, when a SNP is in a CNV or a SD, the two allele frequency measurements are not identical. This is because the usage of genotype-based allele frequencies assumes that every heterozygote has one of each SNP allele, which is not true in case of duplication (eg. 3 C's and 1 A's). The pooled sample-based measurement captures the unequal allelic abundance in heterozygotes.

### II. Computation of conditional probabilities

The second goal is to compute P(CNV|HWD) and P(HWD|CNV), given sample size (number of individuals genotyped) *n*, frequency of allele A of the observed SNP, 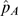, and the significance level for HWD testing, α. HWD is determined by the conventional χ^2^ goodness-of-fit test without continuity correction at α = 0.05 or 0.01. Though it has been proposed that other tests are superior under certain conditions[23], we used the most widely used χ^2^ test, to provide a practical perspective. Four different genotyping error models were tried including 0%, 1%, 5% and 25%. x% genotyping error is defined as follows: x% of heterozygote are read as one of the homozygotes and another x% is read as the other homozygote. This results in excessive homozygotes. Our genotyping error model only misreads heterozygotes as homozygotes, but not vise versa. It is trivial to include the opposite trend, but we do not for the following reasons: an additional genotyping error in the opposite direction would only decrease the overall deviation from HWE by counterbalancing the increased number of observed homozygotes. Experimental techniques often miss one of the two existing alleles, but less often identify an allele that does not exist, unless there is contamination or a high noise level. The 25% genotyping error is not realistic but it provides a comparative perspective.

A procedure for computing the conditional probabilities P(CNV|HWD) and P(HWD|CNV) is described below (See Method S1 for details). Their computation requires knowledge of prior distributions of r, p_1_ and p_2_ and likelihood of 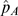 and HWD given r, p_1_, p_2_, n and α.
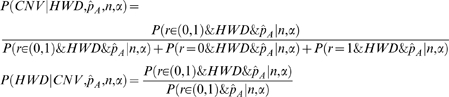



### Prior distributions of allele frequency parameters

The prior distribution of r was set in a hierarchical way. The probabilities of r∈(0,1), r = 0, r = 1 were first set to 14%, 81% and 5%, and within r∈(0,1), the density of r was set to either a beta or a uniform distribution. The beta function parameters were determined so that the mean of r within r∈(0,1) is 0.05.

The joint prior of p_1_ and p_2_ was also set to a hierarchical distribution, so that the probability of being biallelic is reasonably smaller than that of being monomorphic, for each site. Within p_1_∈(0,1) or p_2_∈(0,1), p_1_ and p_2_ are uniformly distributed (details in Method S1).

### Likelihood of 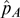 and HWD given r, p_1_ and p_2_

The likelihood was computed based on the likelihood of every possible observed genotype frequency case that corresponds to the observed allele frequency 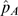. Individual likelihoods were computed based on multinomial function that depends on r, p_1_ and p_2_. HWD was determined for each genotype frequency case using the chi-square test (detail in Method S1).

### Integration of joint probabilities over r, p_1_ and p_2_

In order to approximate the integrals, M independent random samples of triplets (r, p_1_, p_2_) or pairs or singlets were drawn from the prior distribution within (0,1)^3^, (0,1)^2^ or (0,1), respectively. 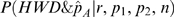 was averaged over these M cases, to compute each of the 15 integrations listed in Method S1. M was larger than 3000 in all cases, but chosen differently for each case, because some of them took longer to converge. The convergences of an example case are shown in Figure S4.

### Parameter space


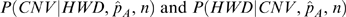
 is computed as described above for a discretized set of 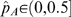, for n = 100 and 1000, at α = 0.05 and 0.01, e_g_ = 0, 0.01, 0.05 and 0.5. Beta and uniform priors for r were tried for comparison. Two independent replicates were generated in order to provide confidence estimates about the probabilities.

### Implementation

All the codes were written in the R language (http://www.r-project.org/).

## Supporting Information

Method S1 PDF file describing method detail.
(0.06 MB PDF)

Figure S1 Range of p1, given r and pooled sample allele frequency. The black diagonal line is the case where the true frequency p1 is identical to the observed frequency. Red and blue lines represent p2 = 0 and p2 = 1, respectively.
(3.64 MB EPS)

Figure S2 The posterior probabilities given HWD computed using the uniform prior, at n = 100, a = 0.05 (left), and n = 1000, a = 0.01 (right), with respect to observed allele frequency. Each row respresents error rate of 0%, 1%, 5% and 25%, from top to bottom, respectively. Estimates are the sample mean of two replicates and the standard deviations are depicted with error bars.
(4.98 MB EPS)

Figure S3 The likelihoods computed using the uniform prior, at n = 100, a = 0.05 (left), and n = 1000, a = 0.01 (right), with respect to observed allele frequency. Each row respresents error rate of 0%, 1%, 5% and 25%, from top to bottom, respectively. Estimates are the sample mean of two replicates and the standard deviations are depicted with error bars.
(4.99 MB EPS)

Figure S4 Convergence of the 15 integrals. The Y values represent joint probabilities (integral multiplied by prior probabilities).
(1.30 MB EPS)
