# Supplementary material for: Quantitative Analysis of Single Nucleotide Polymorphisms within Copy Number Variation
Source: PLoS One. 2008 Dec 18;3(12):e3906. doi: 10.1371/journal.pone.0003906 (PMC2600609; doi:10.1371/journal.pone.0003906)
Supplement: Method S1 — PDF file describing method detail. (0.06 MB PDF) [file pone.0003906.s001.pdf]

Additional file 1 - Method detail

Theoretical derivation of observed genotype frequencies in terms of r, p_1 and p_2

Each observed genotype frequency is a sum of all possible cases that exhibit that particular genotype. For example, $\hat{P}_{AA}$ was computed by summing cases AA/AA, AA/A, AA/-, where the left side of the slash represents the true genotype at the original site and the right side meaning the ectopic site. As an example, the probability of case AA/A was computed as multiplication of three independent events – having AA at the first site, having A at the second site and having two copies in one chromosome and one copy in the other chromosome, which can be expressed as p_1^2, p_2 and 2r(1-r), respectively.

Comutation of conditional probabilities P(CNV|HWD) and P(HWD|CNV)

P(CNV|HWD) and P(HWD|CNV) were computed based on internal allele frequency paramters p_1, p_2 and r, and observed allele frequency($\hat{p}_A$), significance level for HWD testing (α), sample size (n) and genotyping error (e_g). As mentioned in the main text, r, p_1, p_2 refers to true allele frequencies of CNV, SNP at the original site (L1) and SNP at the ectopic site (L2).

$$P(CNV \mid HWD, \hat{p}_A, n, \alpha) = \frac{P(CNV \,\&\, HWD \,\&\, \hat{p}_A \mid n, \alpha)}{P(HWD \,\&\, \hat{p}_A \mid n, \alpha)}$$

$$= \frac{P(r \in (0,1) \,\&\, HWD \,\&\, \hat{p}_A \mid n, \alpha)}{P(r \in (0,1) \,\&\, HWD \,\&\, \hat{p}_A \mid n, \alpha) + P(r = 0 \,\&\, HWD \,\&\, \hat{p}_A \mid n, \alpha) + P(r = 1 \,\&\, HWD \,\&\, \hat{p}_A \mid n, \alpha)}$$

$$P(HWD \mid CNV, \hat{p}_A, n, \alpha) = \frac{P(HWD \,\&\, CNV \,\&\, \hat{p}_A \mid n, \alpha)}{P(CNV \,\&\, \hat{p}_A \mid n, \alpha)} = \frac{P(HWD \,\&\, r \in (0,1) \,\&\, \hat{p}_A \mid n, \alpha)}{P(r \in (0,1) \,\&\, \hat{p}_A \mid n, \alpha)}$$

The numerator and denominator terms were decomposed into a sum of different joint probabilities.

$P(r \in S_r \,\&\, HWD \,\&\, \hat{p}_A \mid n, \alpha) = \sum_{\forall S_{p1}, S_{p2}} P(r \in S_r \,\&\, HWD \,\&\, \hat{p}_A, p_1 \in S_{p_1}, p_2 \in S_{p_2} \mid n, \alpha)$ and

$P(r \in S_r \,\&\, \hat{p}_A \mid n, \alpha) = \sum_{\forall S_{p1}, S_{p2}} P(r \in S_r \,\&\, \hat{p}_A, p_1 \in S_{p_1}, p_2 \in S_{p_2} \mid n, \alpha)$, where S_r, S_{p_1}, S_{p_2} is either $(0,1)$, $\{0\}$ or $\{1\}$. The distributions of r, p_1 and p_2 have a probability mass at 0 or 1 and probability density at (0,1).

In order to compute the probabilities unconditional to r, p_1 and p_2, integration over r, p_1 or p_2 is involved in cases of S_r=(0,1), S_{p_1}=(0,1) or S_{p_2}=(0,1) and the integrals were summed for cases where r, p_1 or p_2 is either 0 or 1. The integrations were performed by sampling randomly from the prior distributions. The computation of joint probabilities $P(r, p_1, p_2, \hat{p}_A \mid n, \alpha)$ and $P(r, p_1, p_2, \hat{p}_A \,\&\, HWD \mid n, \alpha)$ for a given point (r, p_1, p_2) involve computation of $P(\hat{p}_A \mid r, p_1, p_2, n, \alpha)$ and $P(\hat{p}_A \,\&\, HWD \mid r, p_1, p_2, n, \alpha)$. The functions $P(\hat{p}_A \mid r, p_1, p_2, n, \alpha)$ and

$P(\hat{p}_A \,\&\, HWD \,|\, r, p_1, p_2, n, \alpha)$ were computed as described below.

1. Computation of $P(\hat{p}_A \,|\, r, p_1, p_2, n, \alpha)$ and $P(\hat{p}_A \,\&\, HWD \,|\, r, p_1, p_2, n, \alpha)$

Let's denote by x_{AA}, x_{AC}, x_{CC}, the number of individuals with genotype AA, AC and CC, respectively, out of the n samples. For each given $\hat{p}_A$, a set of possible values of (x_{AA}, x_{AC}, x_{CC}) can be determined. The probability distribution of all possible genotype frequencies (x_{AA}, x_{AC}, x_{CC}) is trinomial, which can be expressed as an analytical function of p_1, p_2, r and n. Thus, given the probability mass function G of genotype frequencies, the event $\hat{p}_A$ or $\hat{p}_A$ &HWD is independent of p_1, p_2, r and n. G is defined as:

$$G(x_{AA}, x_{AC}, x_{CC} \,|\, r, p_1, p_2, n) = \frac{n!}{x_{AA}! x_{AC}! x_{CC}!} p_{AA}{}^{x_{AA}} p_{AC}{}^{x_{AC}} p_{CC}{}^{x_{CC}} ,$$

where $p_{AA} = p_1{}^2 (1 - r + rp_2)^2$, $p_{CC} = (1 - p_1)^2 (1 - rp_2)^2$ and $p_{AC}=1-(p_{AA}+p_{CC})$.

With genotyping error rate e_g, $p'_{AA} = p_1{}^2 (1 - r + rp_2)^2 + e_g P_{AC}$, $p'_{CC} = (1 - p_1)^2 (1 - rp_2)^2 + e_g P_{AC}$, and $p'_{AC} = (1 - 2e_g) P_{AC}$ were used instead of p_{AA}, p_{CC} and p_{AC}, respectively. Four different values of e_g, (0, 0.01, 0.05, 0.25) were tried as mentioned above.

For implementation, all possible values of (x_{AA}, x_{AC}, x_{CC}) were deducted for a given value of $\hat{p}_A$. Suppose $T = 2x_{AA} + x_{AC}$. Since $\hat{p}_A = \frac{2x_{AA} + x_{AC}}{2n}$, $T = 2\hat{p}_A n$. Then, T was rounded-off to an integer. Possible x_{AA} values range from 0 to $\frac{T}{2}$ if T is even, or from 0 to $\frac{T-1}{2}$. $x_{AC} = T - 2x_{AA}$, and

$x_{CC} = n + x_{AA} - T$. For each possible combination of (x_{AA}, x_{AC}, x_{CC}), $G(x_{AA}, x_{AC}, x_{CC} \mid r, p_1, p_2, n)$ were computed and HWD testing was performed. HWD testing variable w=1 if (x_{AA}, x_{AC}, x_{CC}) is significantly deviated from HWE in the χ2 test, 0 if not.

Now the conditional probabilities are expressed as:

$$P(\hat{p}_A \;\&\; HWD \mid r, p_1, p_2, n, \alpha) = \sum_{x_{AA}=0}^{t} wG(x_{AA}, T - 2x_{AA}, n + x_{AA} - T \mid r, p_1, p_2, n)$$

$$P(\hat{p}_A \mid r, p_1, p_2, n, \alpha) = \sum_{x_{AA}=0}^{t} G(x_{AA}, T - 2x_{AA}, n + x_{AA} - T \mid r, p_1, p_2, n),$$

where $t = \frac{T}{2}$ if T is even, or $t = \frac{T-1}{2}$ if T is odd.

Chi-square tests were performed without continuity correction. In small-sample cases where χ2 test is unavailable (eg. one of the cells has a value 0), w was set equivalent to HWD. For comparison and to provide evaluation of robustness, we also ran the same program with w=1 for those where chi-square test is not available for n=1000, α=0.01. The results were virtually identical to the results with the w=0 setting (data not shown). An exact test[1] would be more appropriate in these cases, but for computational homogeneity and convenience chi-square tests were used for all cases.

2. Prior distributions

Three prior functions are defined as follows for r, p_1 and p_2: $\pi_r(r)$, $\pi_{p_1 p_2}(p_1, p_2)$ and $\pi_{p_1}(p_1)$:

1) $$\pi_r = \begin{cases} K_{CNV} \cdot beta(r, \kappa = 1, \beta = 19) & if \quad r \in (0,1) \\ K_{reg} & if \quad r = 0 \\ K_{SD} & if \quad r = 1 \end{cases},$$

where $beta(r,\kappa,\beta) = \frac{\Gamma(\kappa+\beta)}{\Gamma(\kappa)\Gamma(\beta)} r^{\kappa-1}(1-r)^{\beta-1}$, where Γ is a gamma function. K_{CNV}, $K_{Regular}$ and K_{SD} represent the proportion of CNV, regular and SD regions in the genome, respectively. They were set to 14%, 81% and 5% each, which are roughly consistent with previous estimates (See introduction). For r=0 or 1, $\pi_r(r)$ has probability mass, that corresponds to the percentage of normal and SD regions in the genome. Otherwise, $\pi_r(r)$ has probability density with priority towards r<0.05, in consistence to the previous knowledge. The beta function parameters κ and β were set as above, so that the mean of r is 0.05. For comparison, a uniform prior was also tried instead of the beta function.

2) $\pi_{p_1 p_2}(p_1, p_2)$ and $\pi_{p_1}(p_1)$

A joint prior distribution for p_1 and p_2 was defined using the rate of a single site becoming polymorphic, ρ. In presence of two duplicate sites, the probability of both sites being polymorphic was assumed to be ρ^2, because it can be considered as two independent SNP-creating events. The case where one of the sites is monomorphic and the other is polymorphic can be regarded as a single event, and its probability is ρ. The case where both sites are monomorphic can also be regarded as a single event, although the evolutionary path must be different. Considering these factors, we modeled our prior of p_1 and p_2 as follows:

$$\pi_{p_1p_2}(p_1,p_2)=\begin{cases}\rho^2 & if \quad p_1\in(0,1), p_2\in(0,1) \quad (X1:SNP, X2:SNP)\\ \rho/2 & if \quad p_1\in\{0,1\}, p_2\in(0,1) \quad (X1:monomorphic, X2:SNP)\\ \rho/2 & if \quad p_1\in(0,1), p_2\in\{0,1\} \quad (X1:SNP, X2:monomorphic)\\ \rho/2 & if \quad (p_1,p_2)\in\{(0,1),(1,0)\} \quad (X1, X2:monomorphic, different)\\ 1-3\rho-\rho^2 & if \quad (p_1,p_2)\in\{(0,0),(1,1)\} \quad (X1, X2:monomorphic, same)\end{cases}$$

When p_2 is not applicable (when r=0),

$$\pi_{p_1}(p_1)=\begin{cases}\mu & if \quad p_1\in(0,1)\\ (1-\mu)/2 & if \quad p_1\in\{0,1\}\end{cases}$$

When p_1 or p_2 is 0 or 1, the prior distribution represents a probability mass, whereas when p_1 or p_2 is in between 0 and 1, it represents a uniform probability density. ρ =1/300 was used, to reflect the current estimate of SNP density (10 millions)[2].

3. Decomposition of integration according to probability mass and density regions.

Since our priors are hierarchical mixtures of mass and density, we divided the cases accordingly and performed integration independently for each. The following table lists the cases and corresponding integral forms. The joint probability $P(r\in S_r \;\&\; \hat{p}_A \;\&\; p_1\in S_{p_1} \;\&\; p_2\in S_{p_2} \mid n,\alpha)$ was computed likewise. In the remaining 12 cases (r=0 & p_1=0, r=0 & p_1=1, p_1=p_2=0, p_1=p_2=1), the likelihood is 0 because they result in

observed monomorphism, when there is no experimental errors or when genotyping errors occur only in the hetero→ homo direction.

S_{p_1}	S_{p_2}	$P(r \in (0,1)\ \&\ HWD\ \&\ \hat{p}_A\ \&\ p_1 \in S_{p_1}\ \&\ p_2 \in S_{p_2} \mid n, \alpha)$
(0,1)	(0,1)	$\int_{r\in(0,1)} \int_{p_1\in(0,1)} \int_{p_2\in(0,1)} P(HWD\ \&\ \hat{p}_A \mid r, p_1, p_2, n) \cdot \pi_r(r)\pi_{p_1p_2}(p_1, p_2) dp_2 dp_1 dr$
(0,1)	0	$\int_{r\in(0,1)} \int_{p_1\in(0,1)} P(HWD\ \&\ \hat{p}_A \mid r, p_1, p_2 = 0, n) \cdot \pi_r(r)\pi_{p_1p_2}(p_1, 0) dp_1 dr$
(0,1)	1	$\int_{r\in(0,1)} \int_{p_1\in(0,1)} P(HWD\ \&\ \hat{p}_A \mid r, p_1, p_2 = 1, n) \cdot \pi_r(r)\pi_{p_1p_2}(p_1, 1) dp_1 dr$
0	(0,1)	$\int_{r\in(0,1)} \int_{p_2\in(0,1)} P(HWD\ \&\ \hat{p}_A \mid r, p_1 = 0, p_2, n) \cdot \pi_r(r)\pi_{p_1p_2}(0, p_2) dp_2 dr$
1	(0,1)	$\int_{r\in(0,1)} \int_{p_2\in(0,1)} P(HWD\ \&\ \hat{p}_A \mid r, p_1 = 1, p_2, n) \cdot \pi_r(r)\pi_{p_1p_2}(1, p_2) dp_2 dr$
0	1	$\pi_{p_1p_2}(0,1) \int_{r\in(0,1)} P(HWD\ \&\ \hat{p}_A \mid r, p_1 = 0, p_2 = 1, n) \cdot \pi_r(r) dr$
1	0	$\pi_{p_1p_2}(1,0) \int_{r\in(0,1)} P(HWD\ \&\ \hat{p}_A \mid r, p_1 = 1, p_2 = 0, n) \cdot \pi_r(r) dr$

S_{p_1}	S_{p_2}	$P(r = 1\ \&\ HWD\ \&\ \hat{p}_A\ \&\ p_1 \in S_{p_1}\ \&\ p_2 \in S_{p_2} \mid n, \alpha)$
(0,1)	(0,1)	$\pi_r(1) \int_{p_1\in(0,1)} \int_{p_2\in(0,1)} P(HWD\ \&\ \hat{p}_A \mid r = 1, p_1, p_2, n) \cdot \pi_{p_1p_2}(p_1, p_2) dp_1 dp_2$
(0,1)	0	$\pi_r(1) \int_{p_1\in(0,1)} P(HWD\ \&\ \hat{p}_A \mid r = 1, p_1, p_2 = 0, n) \cdot \pi_{p_1p_2}(p_1, 0) dp_1$
(0,1)	1	$\pi_r(1) \int_{p_1\in(0,1)} P(HWD\ \&\ \hat{p}_A \mid r = 1, p_1, p_2 = 1, n) \cdot \pi_{p_1p_2}(p_1, 1) dp_1$

0	(0,1)	$\pi_r(1) \int_{p_2 \in (0,1)} P(HWD \,\&\, \hat{p}_A \mid r=1, p_1=0, p_2, n) \cdot \pi_{p_1 p_2}(0, p_2) dp_2$
1	(0,1)	$\pi_r(1) \int_{p_2 \in (0,1)} P(HWD \,\&\, \hat{p}_A \mid r=1, p_1=1, p_2, n) \cdot \pi_{p_1 p_2}(1, p_2) dp_2$
0	1	$\pi_{p_1 p_2}(0,1) \pi_r(1) P(HWD \,\&\, \hat{p}_A \mid r=1, p_1=0, p_2=1, n)$
1	0	$\pi_{p_1 p_2}(1,0) \pi_r(1) P(HWD \,\&\, \hat{p}_A \mid r=1, p_1=1, p_2=0, n)$

S_{p_1}	S_{p_2}	$P(r=0 \,\&\, HWD \,\&\, \hat{p}_A \,\&\, p_1 \in S_{p_1} \,\&\, p_2 \in S_{p_2} \mid n, \alpha)$
(0,1)	-	$\pi_r(0) \int_{p_1 \in (0,1)} P(HWD \,\&\, \hat{p}_A \mid r=0, p_1, (p_2), n) \cdot \pi_{p_1}(p_1) dp_1$

Table S1. Joint probabilities for different cases of S_{p_1} and S_{p_2}.

1. Emigh TH (1980) A comparison of tests for Hardy-Weinberg Equilibrium. Biometrics 36: 627-642.

2. Reich DE, Gabriel SB, Altshuler D (2003) Quality and completeness of SNP databases. Nat Genet 33: 457-458.
